# Piecewise quadratic growth during the 2019 novel coronavirus epidemic

**DOI:** 10.1016/j.idm.2020.08.014

**Published:** 2020-09-11

**Authors:** Axel Brandenburg

**Affiliations:** Nordita, KTH Royal Institute of Technology and Stockholm University, SE-10691 Stockholm, Sweden

**Keywords:** COVID-19, Coronavirus, Epidemic, SIR model, Reaction–diffusion equation

## Abstract

The temporal growth in the number of deaths in the COVID-19 epidemic is subexponential. Here we show that a piecewise quadratic law provides an excellent fit during the thirty days after the first three fatalities on January 20 and later since the end of March 2020. There is also a brief intermediate period of exponential growth. During the second quadratic growth phase, the characteristic time of the growth is about eight times shorter than in the beginning, which can be understood as the occurrence of separate hotspots. Quadratic behavior can be motivated by peripheral growth when further spreading occurs only on the outskirts of an infected region. We also study numerical solutions of a simple epidemic model, where the spatial extend of the system is taken into account. To model the delayed onset outside China together with the early one in China within a single model with minimal assumptions, we adopt an initial condition of several hotspots, of which one reaches saturation much earlier than the others. At each site, quadratic growth commences when the local number of infections has reached a certain saturation level. The total number of deaths does then indeed follow a piecewise quadratic behavior.

## 1 Introduction

The COVID-19 pandemic has attracted significant attention among modelers of the spreading of the disease (Backer et al., 2020; Chen et al., 2020; Liang, Xu, & Fu, 2020; Wang & Zhang, 2020; Wu, Leung, & Leung, 2020; Zhou, Liu, & Yang, 2020). Knowing the evolution of the numbers of cases and fatalities gives important clues about the stage and severity of the epidemic. On theoretical grounds, one expects the number to increase exponentially – at least in the beginning (Britton, 2020a). At the same time, however, control interventions lead to subexponential growth (Chowell et al., 2016; Fenichel et al., 2011; Roosa et al., 2020; Santermans et al., 2016; Tang et al., 2020), but this is harder to quantify and to model.

In connection with COVID-19, it was noticed early on that the increase is close to quadratic (Brandenburg, 2020a; Fukui & Furukawa, 2020; Maier & Brockmann, 2020; Ziff & Ziff, 2020). While this was always thought to be a consequence of the adopted control interventions and confinement efforts, it was soon realized that a quadratic growth can more directly be explained as a consequence of what is called peripheral spreading; see the appendix of version 2 of February 14 of Brandenburg (2020a).

The idea of peripheral growth has caught the interest of modelers in subsequent studies (Blanco et al., 2020; Bodova & Kollar, 2020; Li, Chen, & Deng, 2020; Medo, 2020; Radicchi & Bianconi, 2020; Singer, 2020; Triambak & Mahapatra, 2020; Wu, Darcet, Wang, & Sornette, 2020). Such an interpretation can have far-reaching consequences, because it implies that the spreading of the disease has effectively stopped in the bulk of some confined population. Further spreading is only possible on the periphery, for example through asymptomatic individuals that escaped detection. This inevitably led to further spreading outside China through the rest of the world.

The purpose of the present paper is to substantiate the idea of peripheral growth through standard epidemiological modeling. The simplest of such models is that of Kermack and McKendrick (1927). It is now commonly referred to as the SIR model, where *S* stands for the number of susceptible individuals, *I* for the number of infectious individuals, and *R* for the number of recovered, deceased, or immune individuals. The spatial dimension is added to the problem by introducing a diffusion operator (Källén et al., 1985; Murray et al., 1986; Noble, 1974); see also the text book by Murray (2003) for a detailed account on biological modeling in space and time. We show that this model can explain the piecewise quadratic growth observed during COVID-19 outbreak. The shorter time constant during the second quadratic growth phase is modeled as an increase in the number of separated hotspots, from which peripheral growth occurs. We begin, however, with a detailed discussion of the evidence for quadratic growth during various stages of COVID-19.

## 2 Quadratic versus exponential growth

**Fig. 1:**
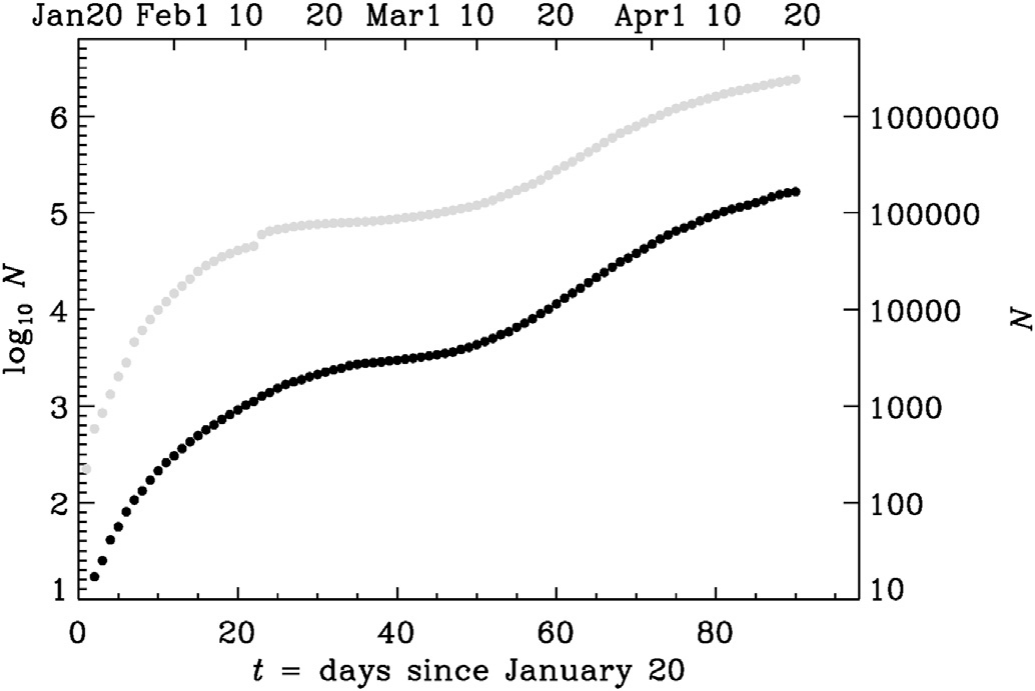
Logarithmic representation (base 10) of the number of deaths (black symbols) and infections (gray symbols) as a function of time since January 20, 2020. The actual date is given on the upper axis and the actual values of *N* are given on the right-hand axis.

Our primary interest is in the number of infections, but this number is uncertain because it depends on the amount of tests that are done in each country. A more robust proxy is the number of deaths. For the data after January 22, 2020, we use the worldometers website,^1^ while the data of earlier days can be found on the DEVEX website.^2^ In Fig. 1, we show the number of infections and the number of deaths in a semi-logarithmic representation. Following Brandenburg (2020a), time is here counted as the number of days after January 20, which he identified as the date when quadratic growth commenced. Specifically, he found

(1) $$N_{\text{fit}}\left(t\right)={\left[\left(t-\text{Jan }20\right)/0.7\;\text{days}\right]}^{2}\;\;\left(\text{quadratic fit}\right)\text{.}$$

This means that every 0.7 days, the square root of *N* changes by one. Such an increase is much slower than an exponential one, where instead the logarithm of *N* changes by one during one characteristic time. To illuminate the quadratic growth in more detail, we consider an example for January 30. In that case, Equation (1) predicted $N_{\text{fit}}={\left(10/0.7\right)}^{2}=204$, so 0.7 days later, $N_{\text{fit}}^{1/2}$ changed by one (from 14.3 to 15.3) and therefore $N_{\text{fit}}={\left(10.7/0.7\right)}^{2}=234$, corresponding to an increase of *N* by 30. On February 20, i.e., 31 days after January 20, the formula predicted $N_{\text{fit}}={\left(31/0.7\right)}^{2}=1960$, so 0.7 days later, $N_{\text{fit}}={\left(31.7/0.7\right)}^{2}=2050$ has increased by 90. This gives us a sense of the way *N* increased. These numbers agree quite well with the actual ones.

**Fig. 2:**
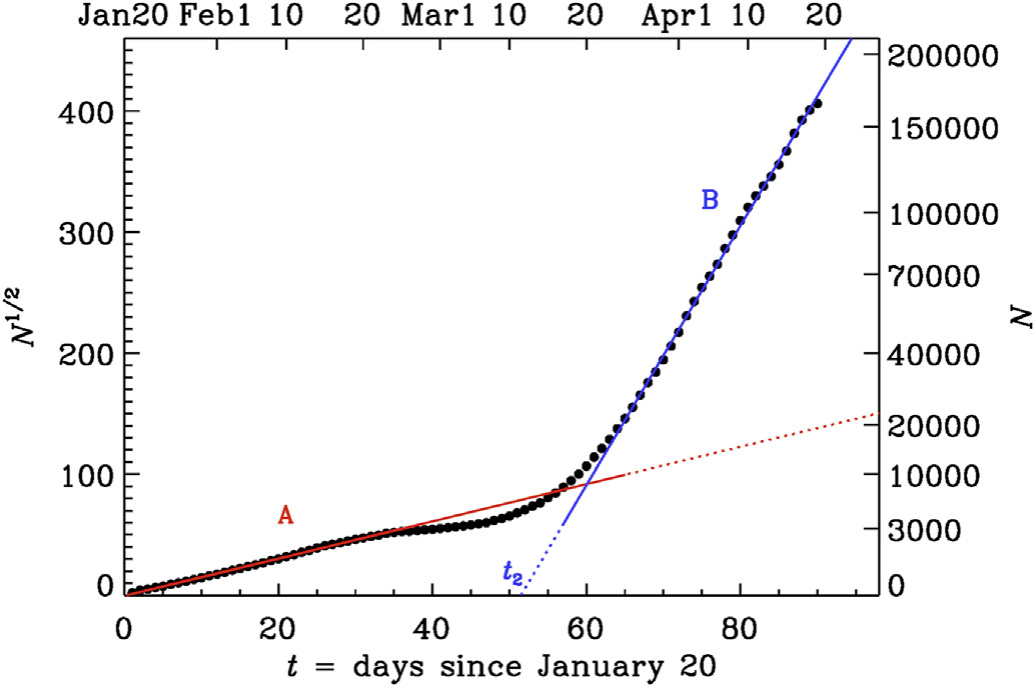
Same as Fig. 1, but for the square root of *N* (black dots). The red line corresponds to the fit given by Equation (1), and the blue line is a similar fit with parameters given in Table 2.

Real data never agree perfectly with any particular mathematical growth law. It is therefore important to quantify the accuracy of any such a description. To assess the accuracy of a description in terms of a quadratic growth law, it is useful to plot the square root of the number of death, $N^{1/2}$, because this quantity then increases linearly in such a representation; see Fig. 2. We immediately notice the appearance of two subranges, A and B, with approximately quadratic growth, but different slopes.

**Fig. 3:**
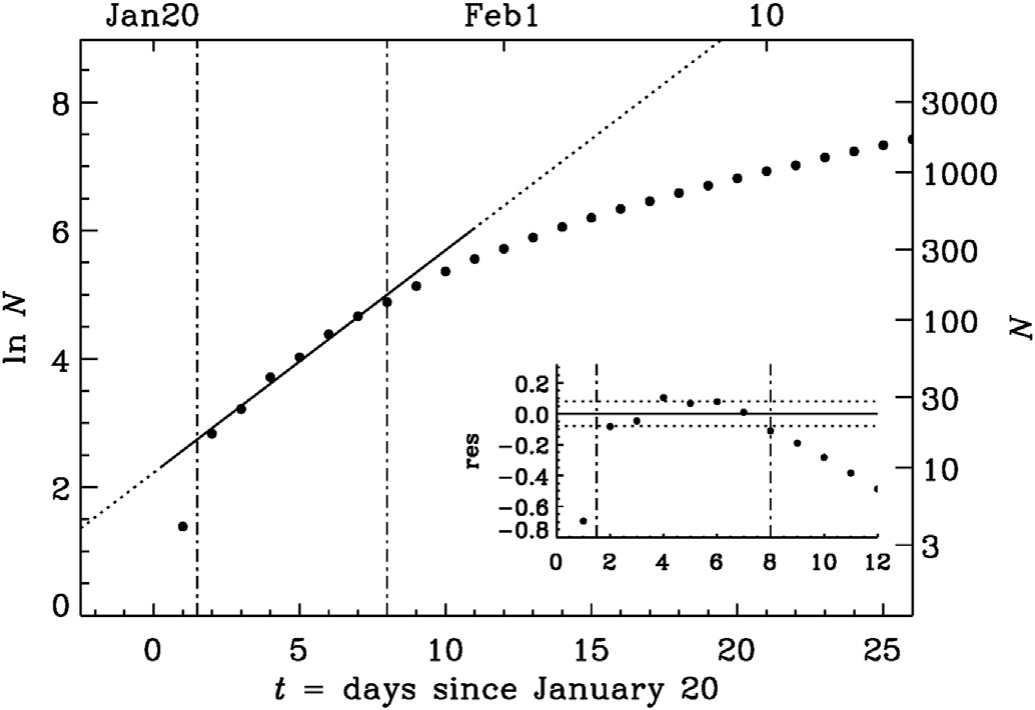
Semi-logarithmic representation of *N* during the early phase. The two vertical dash-dotted line indicate the fit range where lnN increases approximately linearly with *t*. The inset shows the residual with the two horizontal dotted lines indicating the value of ±σ. Here and in the following plots, the natural logarithm is used.

Before substantiating the reality of quadratic growth, we first examine the possibility of exponential growth during early times. In Fig. 3, we show a semi-logarithmic representation for the end of January 2020. We see that, even at early times, there is no convincing evidence for exponential growth, although it is always possible to identify an approximately constant slope during short time intervals. The fit shown in Fig. 3 for January 22–28 is given by

(2) $$N_{\text{fit}}\left(t\right)=\text{exp}\left[\left(t-t_{0}\right)/\tau \right]\;\left(\text{exponential fit}\right)\text{,}$$

where τ is the *e*-folding time and $t_{0}$ is some reference time (where the fit intersects the abscissa); see Table 1 for a summary of the parameters. The *e*-folding time increases from about 3 days during Interval I to about 8 days during Intervals II and III; see the values of τ in Table 1.

**Table 1:** Parameters of three exponential fits. Time is in days, starting on January 20, 2020.

Interval	$t_{1}$	$t_{2}$	$t_{0}$	τ	σ
I	1	8	−6.4	2.9	0.079
II	10	25	−32.7	7.8	0.060
III	54	75	−19.6	8.5	0.021

**Table 2:** Like Table 1, but for the parameters of the square root fits.

Interval	$t_{1}$	$t_{2}$	$t_{0}$	τ	σ
A	5	36	0.3	0.65	0.042
B	65	90	51.5	0.093	0.019

Next, to quantify the accuracy of the fits given by Equation (2) for limited time intervals, $t_{1}\leq t\leq t_{2}$, we compute the relative residual as

(3) $$\text{res}=\left[N\left(t\right)/N_{\text{fit}}\left(t\right)-1\right]\text{.}$$

**Fig. 4:**
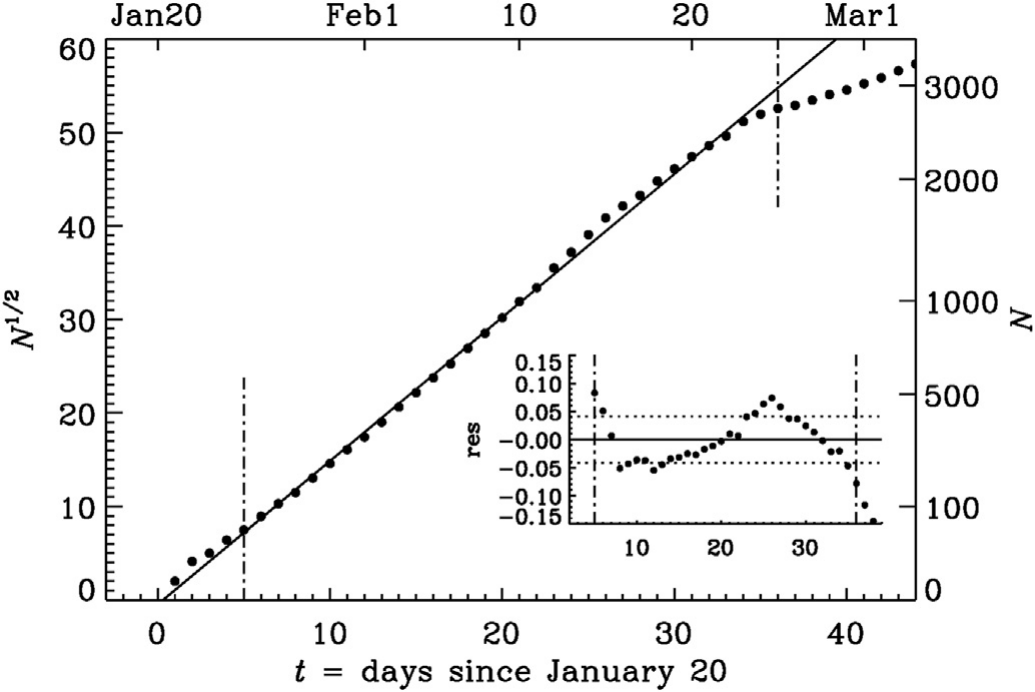
Similar to Fig. 3, but for a square root representation of *N* during the early phase.

This residual is shown as an inset to Fig. 3, and its standard deviation, $\sigma =\langle {\left(\text{res}\right)}^{2}\rangle^{1/2}$, is indicated by dotted lines. Here, angle brackets denote averaging over the time span $t_{1}\leq t\leq t_{2}$. For Interval I, σ is approximately 8%; see Table 1. For the quadratic fit, we write

(4) $$N_{\text{fit}}\left(t\right)={\left[\left(t-t_{0}\right)/\tau \right]}^{2}\;\left(\text{quadratic fit}\right),$$

where τ is again a characteristic time and $t_{0}$ is a reference time, where the fit intersects the abscissa. This is fairly analogous to the exponential fit, but the meaning of τ is different; see Table 2 for the parameters of the quadratic fits. The residual of Equation (3) applies to both types of fits. The quadratic fit of Equation (1) has a standard deviation of only 4% over a much longer time interval of about 30 days in Interval A (see Fig. 4) compared to the exponential fits for Interval I (Fig. 3) and II (see Table 1 for the parameters).

**Fig. 5:**
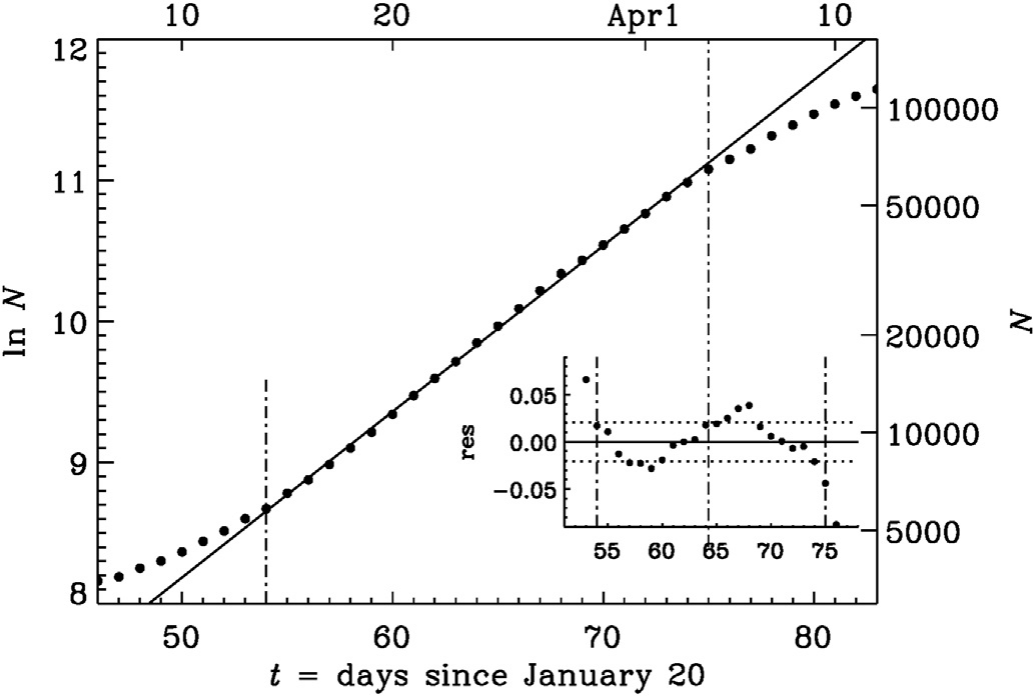
Similar to Fig. 3, but for the late phase (Interval III).

**Fig. 6:**
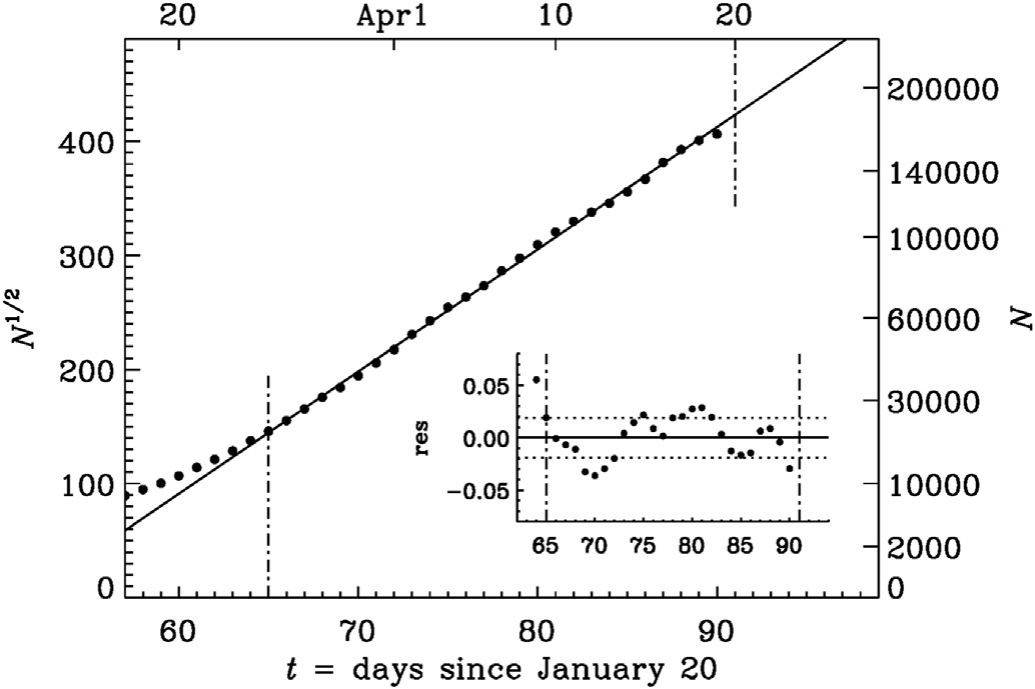
Similar to Fig. 4, but for the late phase (Interval B).

As mentioned before, there is an intermediate phase (Interval III), where the growth is indeed approximately exponential with a value of σ of about 2% for about 20 days; see Fig. 5. This stage is followed by a quadratic growth (Interval B) until the present time with σ=1.9%. In Fig. 6 we show such a representation. The characteristic time is now about 0.09 days, which is eight times shorter than the time during the early stage in Interval A. This means that $N^{1/2}$ changes by one every 0.09 days or by about ten every day. We have to remember that *N* is now larger than for Interval A: when $N^{1/2}=400$ (late times in Fig. 6), a change of $N^{1/2}$ by ten per day corresponds to a change of *N* from $400^{2}=160000$ to $410^{2}=168100$, i.e., a change by about 8000 in one day.

### 3 Heuristic model of peripheral spreading

Contrary to the usual exponential growth, a quadratic growth can be the result of control interventions. It can be explained by spreading on the periphery of a bulk structure, which can be of geometrical or sociological nature (Britton, 2020b). In the bulk, no further infections are possible. At the level of an epidemic model, it would correspond to a situation where the local population density has effectively reached saturation levels in the number of infections (Britton, Ball, & Trapman, 2020). In the case of COVID-19, however, it is more realistic to describe this as a state of partial confinement and isolation of individuals or small groups of people.

**Fig. 7:**
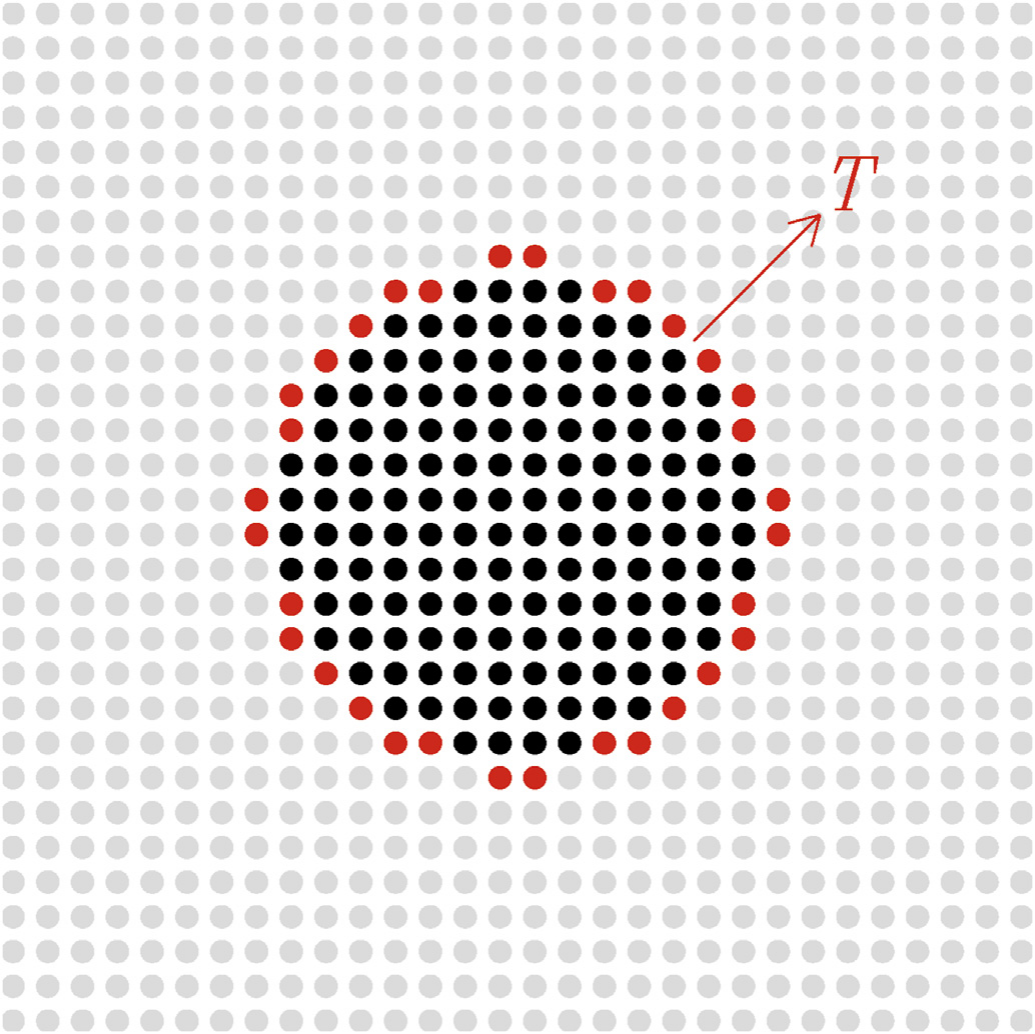
Sketch illustrating the group of infected people confined to the bulk (here for N=148, black filled symbols) and a group of people at the periphery (here $n=32\approx 3\sqrt{N}$, red filled symbols) responsible for spreading the disease with a characteristic time scale *T*.

Here we propose a model where the continued confinement efforts prevent spreading within the bulk of the infected population, but these efforts cannot prevent spreading on the periphery; see Fig. 7 for a sketch. The rate dN/dt, with which *N* increases in time, is therefore equal to the number of infected people on the periphery divided by a characteristic spreading time *T*. We therefore arrive at the following simple differential equation

(5) $$\frac{\text{d}N}{\text{d}t}=\frac{n}{T},$$

where *n* is the number of people in a narrow strip around the periphery. Its size scales with the ratio of the circumference (=2πr for a circle of radius *r*) to the square root of the area ($=\pi r^{2}$), so $n\approx 2\sqrt{\pi N}$. Here, the prefactor depends on the geometry and we would have $n=4\sqrt{N}$ for a rectangular geometry. We may therefore set $n=\alpha \sqrt{N}$, where α≈3.5 for a circular geometry. Inserting this into Equation (5) yields

(6) $$\frac{\text{d}N}{\text{d}t}=\frac{\alpha }{T}\sqrt{N},$$

with the solution

(7) $$N\left(t\right)={\left(\alpha t/2T\right)}^{2}.$$

The empirical analysis of Section 2 suggested a growth of the form $N\left(t\right)={\left(t/\tau \right)}^{2}$, with τ≈0.7days (or about 17 h) and *t* being the time in days after January 20, 2020 for Interval A and τ≈0.093days for Interval B. For a circular geometry, this implies that the spreading times are T=ατ/2=1.2days and 0.16days for Intervals A and B, respectively.

The idea of a geometrically confined bulk with a surrounding periphery may need to be generalized to sociological or network structures that can follow similar patterns (Britton, 2020b; Iannelli et al., 2017; Prasse et al., 2020). In the present work, we do not make any attempts to analyze this aspect further, but refer instead to recent work of Sanche, 2020, who analyzed the spatial patterns of COVID-19 during the early phase, and to the work of Ziff and Ziff (2020), who also discussed spreading on a fractal network.

The increase in the slope of $N^{1/2}$ versus *t* corresponds to a decrease of τ by a factor of about eight for Interval B. This can be the result of multiple separate hotspots, each of which display peripheral growth. This idea will be substantiated further in the following, where we use the spatio-temporal epidemiological model of Noble (1974). Such a model leads to radial expansion waves that propagate at constant speed. It is therefore expected to lead to situations similar to what we have discussed above.

At later times, several hotspots can merge. This leads to a decrease in the slope, which has in fact also been observed since the middle of May. Corresponding plots are presented along with the datasets for the present paper (Brandenburg, 2020b). The discussion of such models will be postponed to a subsequent paper.

## 4 Epidemiological model with spatial extent

The SIR model of Kermack and McKendrick (1927) is a predator–prey type model, where the number of prey corresponds to the susceptible individuals *S*, and the number of predators corresponds to the infected population *I*. The latter also spreads to their neighbors by diffusion, described by a diffusion term $\kappa \nabla^{2}I$ with κ being a diffusion coefficient (Källén et al., 1985; Murray et al., 1986; Noble, 1974). Finally, there is the number of diseased or recovered individuals *R*. The quantity *I* can be identified with the variable *N* used in Section 2. Thus, we have

(8) $$\frac{\partial S}{\partial t}=-\lambda SI,$$

(9) $$\frac{\partial I}{\partial t}=\lambda SI-\mu I+\kappa \nabla^{2}I,$$

(10) $$\frac{\partial R}{\partial t}=\mu I.$$

In a closed domain, the total number of individuals is constant, so ⟨S+I+R⟩=const. We therefore only need to solve Equations (8), (9).

To solve Equations (8), (9), we employ the pencil code,^3^ a publicly available time stepping code for solving partial differential equations on massively parallel architectures (Brandenburg & Dobler, 2010). Spatial derivatives are computed from a sixth-order finite difference formula and the third order Runge–Kutta time stepping scheme of Williamson (1980) is employed. We use $4096^{2}$ mesh points and run the model for about 1200 time units, which takes about 6 min with 1024 processors on a Cray XC40. We fix the time step to be 0.05 and have checked that the solution did not change when the time step is decreased further. The SIR model is implemented in the current version, and also the relevant input parameter files are publicly available (Brandenburg, 2020b).

We solve Equations (8), (9) in a two-dimensional Cartesian domain with coordinates x=(x,y) and periodic boundary conditions. We characterize the domain size *L* by the smallest wavenumber k=2π/L that fits into the domain.

The model has three parameters: the reproduction rate λ, the rate of recovery μ, and the diffusion constant κ. In this model, a certain fraction of *R* could be interpreted as the number of deaths, but this distinction will not be made in the present work. In addition to the three aforementioned parameters, we have the spatial and temporal coordinates, x and *t*. It is convenient to define nondimensional space and time coordinates as $\tilde{x}=kx$ and $\tilde{t}=\lambda t$. This leaves $\tilde{\mu }=\mu /\lambda$ and $\tilde{\kappa }=\kappa k^{2}/\lambda$ as the only nondimensional input parameters that we shall vary. The population number is normalized by the initial number of susceptible individuals, $S_{0}$, so we can define $\tilde{S}=S/S_{0}$, $\tilde{I}=I/S_{0}$, and $\tilde{R}=R/S_{0}$ as the fractional (nondimensional) population densities. We then have $\langle \tilde{S}+\tilde{I}+\tilde{R}\rangle =1$ at all times.

The tildes will from now on be dropped. In practice, this means that we always keep λ=1 and adopt for the domain size L=2π, so k=1.

**Fig. 8:**
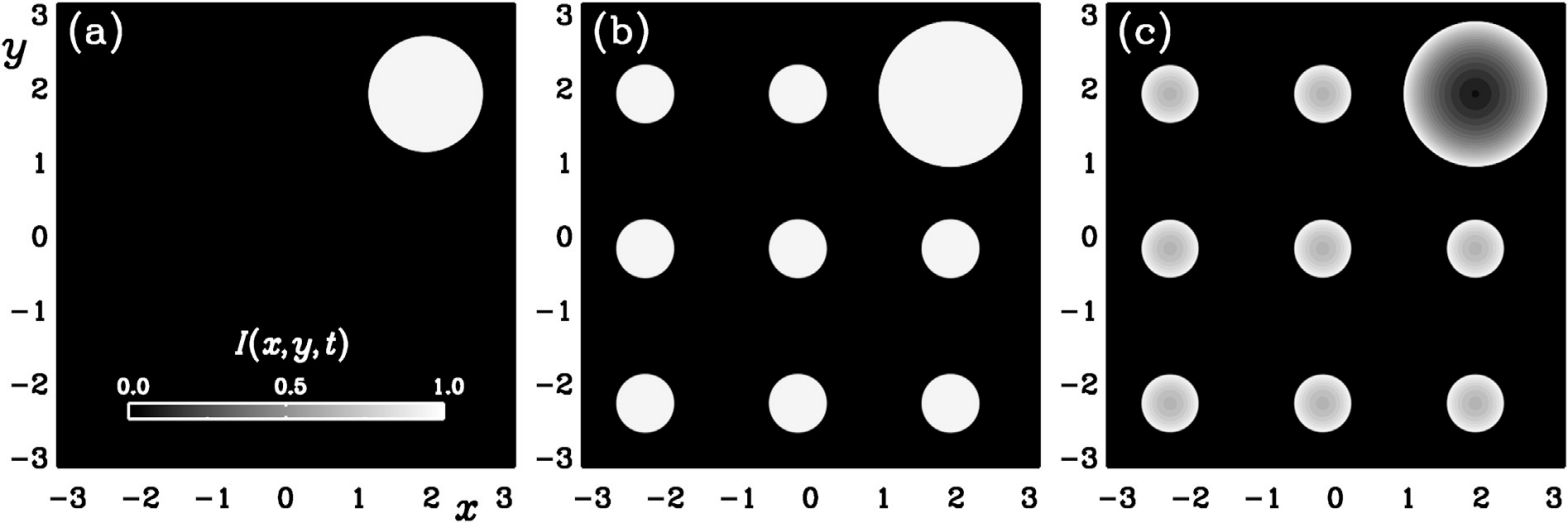
I(x,y,t) for t=400 and μ=0 (a), t=500 and μ=0 (a), and t=500 and $\mu =5\times 10^{-3}$ (c).

As initial condition, we assume S=1 and I=0, except for nine mesh points, where we initialize $I=I_{1}$ on one isolated mesh point and $I=I_{2}$ on eight others. We refer to them as “hotspot”. We always use $I_{1}=10^{-6}$ for the main hotspot, which we place at x≈y≈2; see Fig. 8. Since the growth rate is normalized to unity, one would expect $I_{1}$ to reach unity in a time $t=-\text{ln}10^{-6}=6\times \text{ln}10\approx 14$ in the absence of saturation. We perform different experiments using for the secondary multiple hotspots the values $I_{2}=10^{-60}$, $10^{-180}$, and $10^{-300}$, which, in the absence of saturation, would reach saturation at times t=60×ln10≈140, 180×ln10≈410, and 300×ln10≈700.

We begin by studying models with μ=0, but later we also consider small nonvanishing values of μ. For most of our models, we use $\kappa =10^{-6}$, which is close to the smallest value that is allowed at our resolution of $4096^{2}$. It implies that reaction fronts are sufficiently well resolved. Their size and speed depend on the values of λ and κ and can be obtained on dimensional grounds. It is therefore useful to restore the symbol λ, even though we have already put it to unity. In our case, the width of the front is $\sqrt{\kappa /\lambda }=10^{-3}$. Such an expression is typical of reaction–diffusion equations (Fisher, 1937; Kolmogorov et al., 1937). The front speed is $c=2\sqrt{\lambda \kappa }$ (Murray, 2003), which is characterized by the nondimensional quantity Pe=c/κk=2000, which is also known as the Péclét number.

In Fig. 8 we show gray scale visualizations of I(x,y,t) at two instants shortly before (t=400) and shortly after (t=500) the time when the secondary hotspots become significant (shown here for μ=0). For t=500, we also present a case with μ=0.005=0.5%. We see that this small value of μ hardly affects the spatial spreading of the disease. In the interior of the affected region, however, there is a certain recovery, so I(x,y,t) decreases again at the center of each hotspot. We have not plotted S(x,y,t), but we note that it has an essentially complementary structure in that its value drops locally approximately by the same amount that I(x,y,t) increases.

**Fig. 9:**
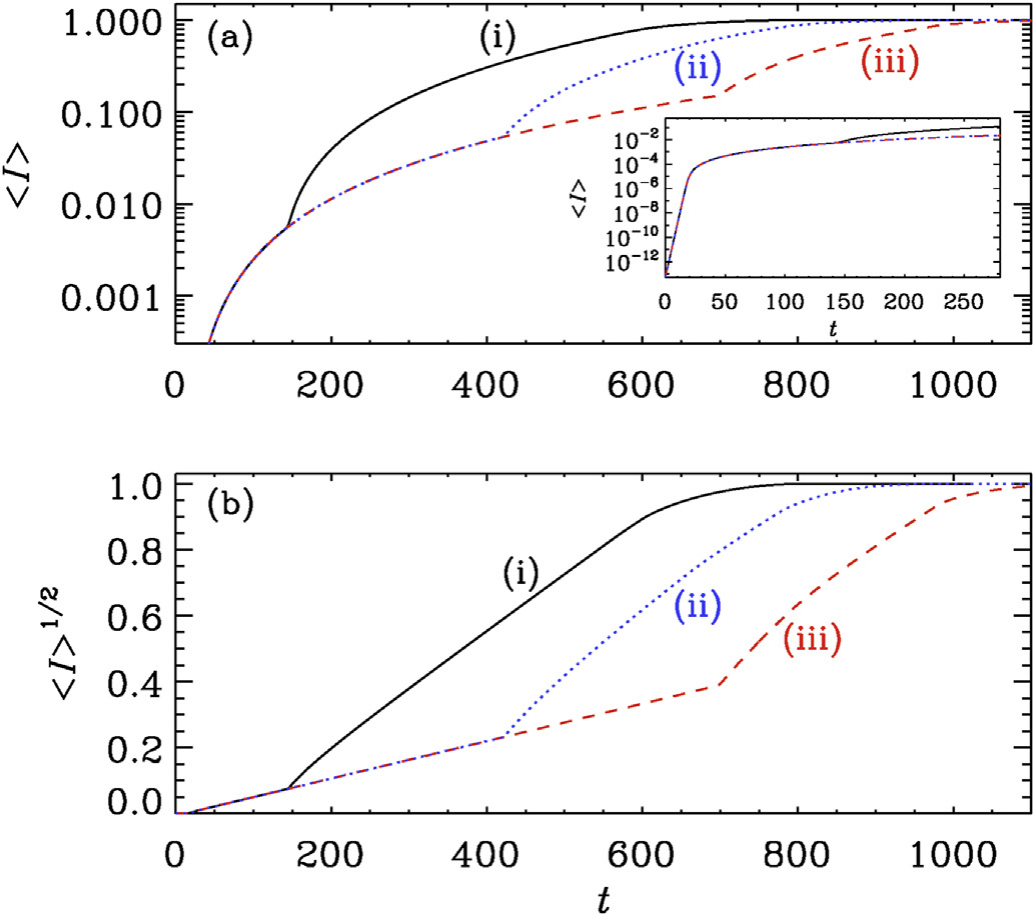
(a) semi-logarithmic and (b) square root representations of ⟨I⟩ for initial values (i) $I_{2}=10^{-60}$, (ii) $10^{-180}$, and (iii) $10^{-300}$.

**Fig. 10:**
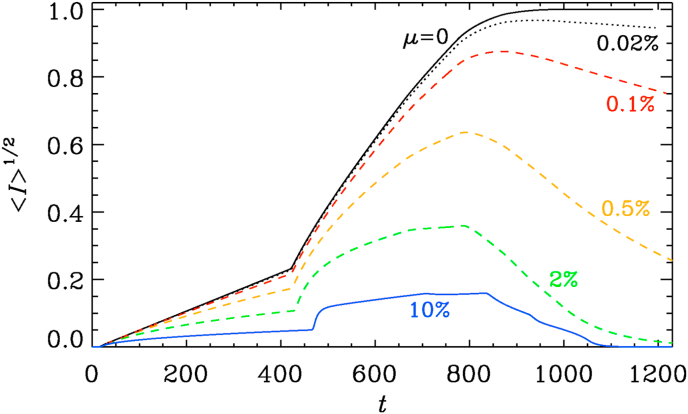
Simulations for μ=0 (black), $\mu =2\times 10^{-4}$ (dotted), $\mu =10^{-3}$ (red), $\mu =5\times 10^{-3}$ (orange), $\mu =2\times 10^{-2}$ (green), and $\mu =10^{-1}$ (blue).

In Fig. 9, we show the evolution of ⟨I⟩ for our three values of $I_{2}$ ($=10^{-60}$, $10^{-180}$, and $10^{-300}$). We see that at very early times, ⟨I⟩ increases exponentially. This is seen in the inset of Fig. 9(a). Locally, the big hotspot has reached saturation within a tiny spot, which then begins to expand. We recall that the primary spot had an initial value of $10^{-6}$, but in the inset we plot the averaged value ⟨I⟩, which can be $4096^{2}\approx 2\times 10^{-7}$ times smaller (see Fig. 10).

At some point, the values of I(x,y,t) at the secondary hotspots begin to become significant and, because of their larger number, begin to dominate the growth of ⟨I⟩. This is similar to the late phase in Interval B shown in Fig. 2. By the argument presented in Section 3, the slope of the graph of $N^{1/2}$, which is proportional to $\langle I\rangle^{1/2}$, should scale with the ratio of their total circumference to the square root of the total area. Therefore, the slope should scale with the square root of the number of secondary hotspots. In this connection, we note that a dependence of the total reaction speed on the number of topologically disconnected regions is also typical of other reaction–diffusion equations and has been seen before; see Fig. 4 of Brandenburg and Multamäki (2004).

Next, we study the effects of changing of μ. We see that already rather small values of around $\mu =2\times 10^{-4}=0.02\text{\%}$ have a noticeable effect at late times, so that ⟨I⟩ reaches a maximum as a function of time at around t=800. The position of this maximum depends only weakly on the value of μ.

**Fig. 11:**
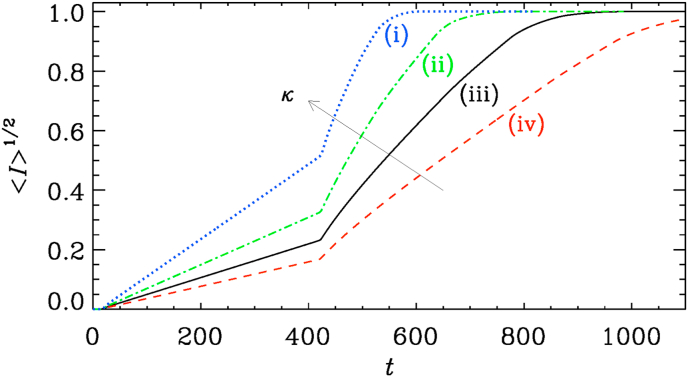
Simulation for $\kappa =5\times 10^{-6}$ (i), $\kappa =2\times 10^{-6}$ (ii), $\kappa =10^{-6}$ (iii), and $\kappa =5\times 10^{-7}$ (iv). The arrow points in the direction of increasing values of κ.

Finally, we study the effects of changing the diffusivity κ. The result is shown in Fig. 11. We see that the speed of spreading, which is roughly $2\sqrt{\lambda \kappa }$, increases with increasing diffusivity.

Having now gained some experience with this model, we can ask what would be realistic parameters related to COVID-19. Given that the slope of $N^{1/2}\left(t\right)$ depends on the value of κ, one might be able to give some estimates. Using the slopes seen in Fig. 11, we find that $\tau^{-1}=\beta \sqrt{\lambda \kappa k^{2}}$, where $\beta_{\text{A}}\approx 0.56$ for Interval A and $\beta_{\text{B}}\approx 2.8$ for Interval B, with the subscript denoting the interval. Again, we have restored here the symbol λ, even though λ=1 was assumed in all of our simulations. The ratio of the two coefficients is around five, which is nearly twice as much as the square root of the number of secondary hotspots, which was expected based on the heuristic argument presented in Section 3. To estimate the effective value of κ, the largest uncertainty comes from the value of *k*, which is the inverse domain size which, in turn, is ultimately related to the size of the affected continents on the Earth. Assuming that $\kappa \approx {\left(\lambda \tau^{2}k^{2}\right)}^{-1}$, we see that with $k\approx {\left(1000\;\text{km}\right)}^{-1}$, $\lambda ={\left(10\;\text{days}\right)}^{-1}$, and τ=1day, we have $\kappa \approx 10^{9}{\text{km}}^{2}/\;\text{day}$, which is much larger than the diffusion coefficient estimated for the spreading of the Black Death in 1347, for which a diffusion coefficient of the order of $10^{2}{\text{km}}^{2}/\;\text{day}$ has been estimated (Noble, 1974).

## 5 Discussion

The present work has demonstrated that for the COVID-19 epidemic, the available data are accurate enough to distinguish between an early exponential growth, as was found for the 2009 A/H1N1 influenza pandemic in Mexico City (Chowell et al., 2016) and the quadratic growth found here. It was expected that the growth would not continue to be exponential, and that it would gradually level off in response to changes in the population behavior and interventions (Fenichel et al., 2011), as found in the 2014–15 Ebola epidemic in West Africa (Santermans et al., 2016). However, that the growth turns out to be quadratic to high accuracy already since January 20 is rather surprising and has not previously been predicted by any of the recently developed models of the COVID-19 epidemic (Chen et al., 2020; Liang, Xu, & Fu, 2020; Zhou, Liu, & Yang, 2020). It is therefore important to verify the credibility of the officially released data; see Robertson et al. (2019) for similar concerns in another context.

In the case of COVID-19, it is remarkable that the fit isolates January 20 as a crucial date in the development of the outbreak. At that time, the actual death toll was just three and the number of confirmed infections just a little over 200.

It is rare that an epidemic provides us with data having such systematic trends as in the present case. One might wonder why this quadratic growth is not generally discussed in the literature. There is a large variety of theoretical models; see Chowell et al. (2016) for a review. Several such models have already been adapted to the COVID-19 epidemic (Britton, 2020a; Chen et al., 2020; Liang, Xu, & Fu, 2020; Zhou, Liu, & Yang, 2020). Furthermore, the idea of control interventions has been discussed in detail (Fenichel et al., 2011), but the present epidemic provides us with an unprecedentedly rich data record with large numbers of infections occurring on an extremely short timescale. All this contributes to having made the quadratic growth so apparent.

By the time the quadratic growth law commenced on January 20, the city of Wuhan was already under quarantine. This suggests that the following thirty days of nearly perfectly quadratic growth where solely the result of human interventions, and therefore potentially highly unstable. This is evidenced by the subsequent period of short exponential growth, before the second period of quadratic growth commenced. The present work has demonstrated that quadratic growth laws are generally the result of partial confinement and that the maximum possible infection levels, given the existing confinement measures, have already been reached. At present, the consequences of relaxing these measures cannot be easily predicted, given that the almost universal lockdown in Europe and the US has been unique in human history.

## Declaration of competing interest

The author claims no conflict of interests.

## Footnotes

1 http://www.worldometers.info/coronavirus/.

2 http://www.devex.com/news/2019-ncov-outbreak-a-timeline-96396.

3 http://github.com/pencil-code, https://doi.org/10.5281/zenodo.2315093.
